# Conditional Synaptic Vesicle Markers for *Drosophila*

**DOI:** 10.1534/g3.118.200975

**Published:** 2019-01-11

**Authors:** Jessica L. Williams, Harold K. Shearin, R. Steven Stowers

**Affiliations:** Department of Cell Biology and Neuroscience, Montana State University, Bozeman, MT

**Keywords:** synaptic vesicle, Drosophila, conditional, fluorescent, epitope

## Abstract

The release of neurotransmitters from synaptic vesicles (SVs) at pre-synaptic release sites is the principle means by which information transfer between neurons occurs. Knowledge of the location of SVs within a neuron can thus provide valuable clues about the location of neurotransmitter release within a neuron and the downstream neurons to which a given neuron is connected, important information for understanding how neural circuits generate behavior. Here the development and characterization of four conditional tagged SV markers for *Drosophila melanogaster* is presented. This characterization includes evaluation of conditionality, specificity for SV localization, and sensitivity of detection in diverse neuron subtypes. These four SV markers are genome-edited variants of the synaptic vesicle-specific protein Rab3. They depend on either the B2 or FLP recombinases for conditionality, and incorporate GFP or mCherry fluorescent proteins, or FLAG or HA epitope tags, for detection.

Synaptic vesicles (SVs) are the foundation of action potential driven chemical synaptic transmission, the major form of neuronal communication in the nervous system (Katz 1971; Jahn and Südhof 1994). The ability to precisely locate synaptic vesicles within a neuron is thus important for understanding how a given neuron transmits information to others and ultimately how neural circuits contribute to the generation of behavior. Knowledge of the subcellular location of SVs in a neuron provides information about where neurotransmitter release can occur and also gives insight into which neighboring neurons may or may not be receiving neurotransmitter-based information. It is thus desirable to have a conditional SV marker that accurately reflects the distribution of SVs in individual neurons in order to understand neuronal signaling and the mechanisms by which behavior is generated.

Previous approaches to localizing SVs in Drosophila have utilized binary transcription systems in which a tagged version of a SV-specific protein is placed under the control of a regulatory element (UAS, LexAop, QUAS) in a responder transgene (Zhang *et al.* 2002; Petersen and Stowers 2011; Nern *et al.* 2015) whose expression is controlled by a transcription factor (GAL4, LexA, QF) in a driver transgene. Although this approach has been useful, it has the potential for inaccuracy because the level of expression of the SV marker is dependent on the strength of the driver and strong drivers may result in expression of the tagged SV protein at a level that exceeds the capacity of a neuron to localize the tagged protein to SVs. Binary transcription system-dependent SV markers are thus potentially subject to erroneous results.

With the development of CRISPR/Cas9 genome editing in Drosophila (Bassett *et al.* 2013; Gratz *et al.* 2013; Ren *et al.* 2013; Yu *et al.* 2013), it is now possible to engineer tagged variants of any gene of interest at their endogenous genomic location such that their expression is under the control of their own regulatory region in a conditional manner. Since a gene’s complete regulatory region is preserved on such a genome-edited chromosome, conditionally-expressible genes will recapitulate temporal and spatial expression in the native cellular pattern of the endogenous gene and, critically for accurate subcellular protein localization, at endogenous levels. This strategy has been successfully used for the SV-specific vesicular acetylcholine transporter (vAChT) by fusing an HA epitope tag onto the vAChT protein and incorporating an upstream transcription stop cassette flanked with FLP recombinase target sites (FRTs) (Pankova and Borst 2017). Although conditional *HA-vAChT* is useful for determining whether a neuron is cholinergic and accurately marking SVs in cholinergic neurons, it has the limitation that it cannot be used to mark SVs in neurons that use neurotransmitters other than acetylcholine.

Here a set of four conditional Drosophila SV markers is described and characterized that are based on the neurotransmitter-independent SV-specific protein Rab3. These conditional SV markers utilize the fluorescent tags GFP and mCherry, the epitope tags FLAG and HA, and utilize the B2 and FLP recombinases for conditionality. Their conditionality, SV specificity, and sensitivity are demonstrated in four diverse types of neurons: the larval neuron Basin-4, the adult lamina neuron L2, adult mushroom body PPL1-α3 neurons, and adult mushroom body Kenyon cell γ neurons.

## Materials and Methods

### Plasmid construction

The *pCFD4-Rab3A* and *pCFD4-Rab3B* double guide RNA plasmids were generated as previously described (Port *et al.* 2014). Targeting sequences included in *pCFD4-Rab3A* guide RNAs are acatgtttgataccgcat and ttctacacatctcaacagca. Targeting sequences included in *pCFD4-Rab3B* guide RNAs are acatgtttgataccgcat and aaatcaatgtgtcagctttc. Donor plasmids were constructed with NEBuilder HiFi (New England Biolabs) in the vector *pHSG298* (Takara Biosciences). The complete sequences of all donor plasmids are shown in Supplemental information.

The *20XUAS-DSCP-B2*, *20XUAS-DSCP-6XGFP*, and *N-syb-GAL4* expression clones were assembled using Gateway MultiSite cloning as previously described (Petersen and Stowers 2011). The *L1-20XUAS-DSCP-L5* entry clone was generated by using the *L1-20XUAS-L5* entry clone (Petersen and Stowers 2011) as template such that the hsp70 minimal promoter was replaced with the Drosophila Synthetic Core Promoter (DSCP) (Pfeiffer *et al.* 2008). The *L5-B2-L2* entry clone used *pJFRC153-20XUAS-IVS-B2*::*PEST* (Nern *et al.* 2011) (Addgene #32134) as template for the B2 recombinase coding sequence that includes a syn21 translation enhancement sequence and omission of the PEST sequence. *UAS-DSCP-FLP* was a gift from the lab of Brian McCabe (EPFL Mind Brain Institute, Lausanne, Switzerland). FLP recombinase with Aspartic acid at position 5 (Nern *et al.* 2011) was PCR amplified from *pJFRC150-20XUAS-IVS-Flp1*::*PEST* (Addgene #32132) to include a syn21 sequence and remove the PEST sequence. It was then transferred into *pBID-UAS-G* (Wang *et al.* 2012) using standard gateway cloning.

A list of expression clones described in this study and the components of the Gateway MultiSite reactions are shown in Table 1. The following entry clones were previously described: *L5-eGFP-L4*, *R4-eGFP-R3*, *L3-4XeGFP-L2* (Shearin *et al.* 2014), *L1-N-syb 5′ Reg-L5* (Shearin *et al.* 2013) and *L5-GAL4-L2* (Petersen and Stowers 2011). The destination vector *pDESTp10aw* was previously described (Shearin *et al.* 2013).

**Table 1:** Original Fly Strains

Genome-edited fly strains	
*B2RT-STOP-B2RT-2XHA-Rab3*	
*B2RT-STOP-B2RT-GFP-Rab3*	
*B2RT-STOP-B2RT-mCherry-Rab3*	
*FRT-F3-3XFLAG-Rab3*	
*FRT-F3-2XHA-Rab3*	
**Transgenic fly strains**	**Landing site**
*UAS-DSCP-FLP*	*VK20*
*UAS-DSCP-B2*	*VK18, JK65C*
*UAS-DSCP-6XGFP*	*VK5*
*N-syb-GAL4*	*JK66B*

### Genome editing

The *pCFD4-Rab3A* and *pCFD4-Rab3B* guide RNA plasmids were co-injected with donor plasmids into embryos of strain *nos-Cas9 TH_attP2* (Ren *et al.* 2013) by Bestgene, Inc. The surviving adults that were injected as embryos were crossed to a 2^nd^ chromosome balancer stock. ∼200 F_1_ progeny males from the first balancer cross were crossed to the 2^nd^ chromosome balancer stock a second time in pools of five males to 10-15 balancer females/vial. After five days of mating, pools of the five males from each vial were subjected to genomic DNA extraction. Pools containing genome-edited chromosomes were identified using a genomic PCR genotyping strategy that paired internal primers unique to the donor constructs (*i.e.*, STOP cassette, GFP, or mCherry) with genomic primers external to the donor constructs on both ends. Pairing primers internal and external to the donor construct in the screening strategy avoids false-positives in which the donor construct has inserted at a spurious location in the genome other than the Rab3 locus. 10-15 F_2_ males from pools that were positive on both ends for DNA fragments of the predicted sizes for successful genome editing were individually crossed to *yw*; *UAS-DSCP-B2*; *N-syb-GAL4* (GFP-Rab3, mCherry-Rab3), or *yw*; *N-syb-GAL4*, *UAS-FLP* (3XFLAG-Rab3, 2XHA-Rab3) in test crosses and third instar larva of the appropriate genotype were screened by immunostaining. Balanced fly stocks containing the desired genome-edited chromosomes were established from the progeny of the test crosses that produced positive immunostains.

### Genomic DNA extraction/PCR genotyping

Five flies were placed in a 1.5ml microfuge tube and homogenized with a pestle (VWR-47750-354) in 10mM Tris pH 8.0, 60mM NaCL, 10mM EDTA, 200μg/ml Proteinase K. After homogenization, tubes were placed in a 37° water bath for 30 min. Samples were subsequently phenol/chloroform extracted and ethanol precipitated. After a 70% EtOH wash, pellets were resuspended overnight in 15μl water. 1μl of purified DNA was used as template in 20μl PCR reactions using KAPA2G polymerase ReadyMix (KAPA Biosystems) as per the manufacturer’s instructions.

### Immunostaining

Larval and adult immunostaining were performed as previously described (Certel and Thor 2004; Petersen and Stowers 2011). Primary antibodies and dilution factors: The dCSP-2 (6D6) mAb 1:50 developed by S. Benzer (Zinsmaier *et al.* 1994), Brp (Nc82) mAb 1:50 developed by E. Buchner (Wagh *et al.* 2006); and SYN (3C11) mAb 1:50 developed by E. Buchner (Klagges *et al.* 1996) were obtained from the Developmental Studies Hybridoma Bank, created by the NICHD of the NIH and maintained at The University of Iowa, Department of Biology, Iowa City, IA 52242; Rabbit DSYT2 anti-Syt 1:500 (Littleton *et al.* 1993); Rat anti-HA 3F10 (Sigma-Aldrich) 1:100; Rabbit anti-HA RM305 (RevMab) 1:500, Rabbit Abfinity anti-GFP (Thermo-Fisher) 1:500, Mouse anti-GFP 3E6 (Thermo-Fisher) 1:200; Rat anti-mCherry 16D7 (Thermo-Fisher) 1:500, Rabbit anti-mCherry (Abcam ab213511) 1:500, Rat anti-FLAG L5 (Covalab) 1:200. Jackson Immunoresearch secondary antibodies: Donkey anti-Ms Alexa 488 (715-546-151), Donkey anti-Rb Alexa 488 (711-546-152), Donkey anti-Rt Alexa 488 (712-546-153), Donkey anti-Ms Cy3 (715-166-151), Donkey anti-Rb Cy3 (711-166-152), Donkey anti-Rt Cy3 (712-166-153), Donkey anti-Ms Alexa 647 (714-606-151), Donkey anti-Rb Alexa 647 (711-606-152), Donkey anti-Rt Alexa 647 (712-606-153). All secondary antibodies were used at a 1:400 dilution.

### Germline excisions and inversions

Germline excisions and inversions were generated by crossing the conditional tagged Rab3 variants to *yw*; *nos-GAL4*; *UAS-DSCP-B2* (*B2RT-STOP-B2RT-GFP-Rab3* and *B2RT-STOP-B2RT-mCherry-Rab3*) or *yw*; *nos-GAL4*; *UAS-FLP* (*FRT-F3-3XFLAG-Rab3* and *FRT-F3-2XHA-Rab3*). Progeny males of the appropriate genotype were crossed to a 2^nd^ chromosome balancer stock to generate potential germline excisions or germline inversions. Germline excisions or inversions were identified by taking individual males from the first balancer cross, crossing them to the 2^nd^ chromosome balancer stock, and screening the third instar larva of the appropriate genotype directly for fluorescence (*GFP-Rab3* and *mCherry-Rab3*) or by immunostaining (*3XFLAG-Rab3* and *2XHA-Rab3*). Balanced fly stocks containing the desired germline excised or inverted chromosomes were established from progeny of positive single male crosses.

### Fly strains

Stocks obtained from the Bloomington Drosophila Stock Center (NIH P40OD018537) were used in this study. Previously described fly strains: *UAS-FLP* (Duffy *et al.* 1998) (BDSC # 4540), *N-syb-GAL4* (Bushey *et al.* 2009), Basin-4: *R72F11AD*; *R57F07DBD* (Ohyama *et al.* 2015), L2: *R53G02AD*; *R29G11DBD* (Tuthill *et al.* 2013), MB131B: *R13F02AD*; *R89B01DBD*, and MB060B: *R82C10AD;R72B05DBD* (Aso *et al.* 2014), *FRT-STOP-FRT-HA-vAChT* (Pankova and Borst 2017) (BDSC #76021), *UAS-CD8-mCherry* (F. Schnorrer, BDSC# 27392), *nos-GAL4* (Tracey *et al.* 2000), *rab3^rup^* (Graf *et al.* 2009) (BDSC# 78045); *UAS-N-syb-GFP* (BDSC# 6922) and *UAS-Syt-GFP* (BDSC# 6926) (Zhang *et al.* 2002). Fly strains original to this study (Table 2): *B2RT-STOP-B2RT-2XHA-Rab3*, *B2RT-STOP-B2RT-GFP-Rab3*, *B2RT-STOP-B2RT-mCherry-Rab3*, *FRT-F3-3XFLAG-Rab3*, *FRT-F3-2XHA-Rab3*, *UAS-DSCP-B2*, *UAS-DSCP-FLP*, *20XUAS-DSCP-6XGFP*, *N-Syb-GAL4*.

**Table 2:** Expression Clone Components

	Component Entry Clones for				
Expression clone	Gateway MultiSite LR reactions				Destination vector
*20XUAS-DSCP-B2*	*L1-20XUAS-DSCP-R5*	*L5-B2-L2*			*pDESTp10aw*
*20XUAS-DSCP-6XGFP*	*L1-20XUAS-DSCP-R5*	*L5-eGFP-L4*	*R4-eGFP-R3*	*L3-4XeGFP-L2*	*pDESTp10aw*
*N-syb-GAL4*	*L1-N-syb 5′ Reg-R5*	*L5-GAL4-L2*			*pDESTp10aw*

### Data availability

Complete sequences of entry clones are available upon request. Complete sequences of donor plasmids are shown in Supplemental Information. Fly strains original to this publication will be deposited at the Bloomington Drosophila stock center or will be made available upon request. Entry clones and donor plasmids will be deposited at Addgene or will be made available upon request. Supplemental material available at Figshare: https://doi.org/10.25387/g3.7575392.

## Results

### Strategic design of conditional tagged Rab3 variants

**Figure 1:**
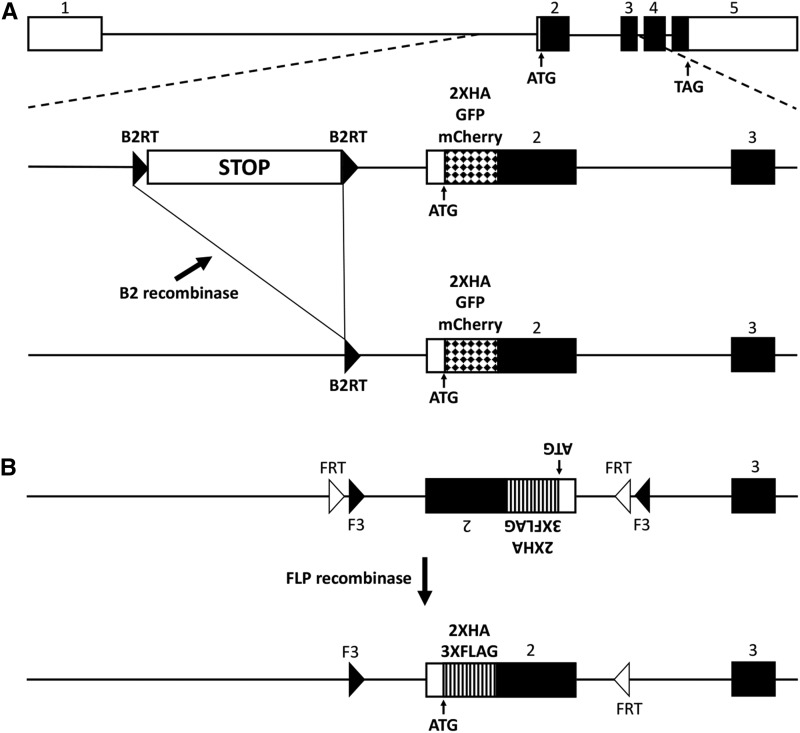
Strategies for generating conditional tagged variants of a Drosophila Rab3 synaptic vesicle marker. A) *top*: genomic exon structure of Drosophila *Rab3.* *middle*: strategy 1 involves incorporating a B2 recombinase target (*B2RT*)-flanked transcription STOP cassette upstream of exon 2 and either a 2XHA epitope tag, or a GFP or mCherry fluorescent protein tag, at the amino-terminus of Rab3. Prior to excision of the transcription STOP cassette, the tagged variants of Rab3 will not be expressed. *bottom*: after B2 recombinase expression in neurons of interest, the tagged variants of Rab3 will be expressed in neurons in which the STOP cassette has been excised. B) *top*: strategy 2 involves paired *FRT* and *F3* FLP recombinase target sites flanking an inverted exon 2 containing 2XHA or 3XFLAG amino-terminal epitope tags. *bottom*: after FLP recombinase expression in neurons of interest and an odd number of inversions followed by an excision, the tagged variants of Rab3 will be stably expressed. Note that FLP recombinase is functional with *FRT* pairs and *F3* pairs but is not functional in an *FRT-F3* combination.

In an effort to develop a conditional synaptic vesicle marker for Drosophila, two CRISPR/Cas9 gene editing strategies were utilized that both target the endogenous locus of Rab3, a gene whose encoded protein is firmly established to localize specifically to SVs in a variety of species (Fischer Von Mollard *et al.* 1990; Südhof 2004; Takamori *et al.* 2006), including Drosophila (Graf *et al.* 2009). In the first strategy, donor constructs were assembled in which a transcription STOP cassette flanked by B2 Recombinase Target sites (B2RTs) (Nern *et al.* 2011) was inserted upstream of the ATG start codon in the first intron of the Rab3 gene (Figure 1A, middle). Three variants of this donor construct were generated all of which include the STOP cassette, but which differ in their incorporation of either a 2XHA epitope tag, or GFP or mCherry fluorescent proteins at the Rab3 amino-terminus. In this strategy, prior to excision of the STOP cassette, transcription of the tagged variants of Rab3 is terminated before the ATG translation start codon and no tagged Rab3 protein is produced. However, after excision of the STOP cassette resulting from selective expression of the B2 recombinase in neurons of interest (Figure 1A, bottom), transcription proceeds normally and the tagged versions of the Rab3 protein are expressed.

In the second approach, donor constructs were assembled such that two strategically oriented *FRT-F3* pairs (Schnütgen *et al.* 2003; Fisher *et al.* 2017) flank an inverted second intron of the Rab3 gene in which the coding sequence of either the 3XFLAG or 2XHA epitope tags was inserted at the Rab3 amino-terminus (Figure 1B, top). *F3* is a variant of the FLP Recombinase Target (*FRT*) site and both *F3* and *FRT* efficiently function as substrates for the FLP recombinase with themselves, but not with each other (Schlake and Bode 1994). Since the second coding intron of Rab3 is initially in opposite orientation to the direction of Rab3 transcription, no epitope-tagged Rab3 protein is produced prior to FLP recombinase-catalyzed inversion. However, after inversion of the second intron resulting from selective expression of FLP recombinase in neurons of interest (Figure 1B, bottom), epitope-tagged versions of Rab3 protein are expressed. Note that in this strategy multiple inversions can occur, alternating between the epitope-tag expressed orientation and non-expressed orientation, but after an odd number of inversions and, inevitably, a single excision, the expressed orientation will become stable.

### Conditionality assessment

Five fly strains, each incorporating at the endogenous Rab3 locus one of the variations described above, were established via CRISPR/Cas9 genome editing (Ren *et al.* 2013). As the goal of this work was to generate conditional tagged variants of Rab3, each of the five fly strains was first assessed for constitutive, or “leaky”, expression. For this assessment, ventral nerve cord (VNC) immunostains of third instar larva with and without pan-neuronal excision of the STOP cassette were compared. Unexpectedly, for *B2RT-STOP-B2RT-2XHA-Rab3,* there was almost as much HA VNC signal without STOP cassette excision (Figure S1A_2_) as with pan-neuronal STOP cassette excision (Figure S1A_3_). No signal was detected in negative control larva (Figure S1A_1_), indicating the observed signal in the experimental larva is coming from 2XHA-Rab3 and not an endogenous antigen recognized by the primary or secondary antibodies. Sequencing of PCR products using DNA from the *B2RT-STOP-B2RT-2XHA-Rab3* fly strain as template revealed that the flanking *B2RTs* and the STOP cassette were complete and mutation-free, thus ruling out a defective STOP cassette as an explanation for its ineffectiveness in terminating transcription. The constitutive expression of 2XHA-Rab3 is particularly enigmatic given the fact that the GFP-Rab3 and mCherry-Rab3 versions show only a trace of leak (see below) and the placement of the STOP cassette is at the identical nucleotide position in the Rab3 gene in all three versions. Due to its high level of constitutive expression, *B2RT-STOP-B2RT-2XHA-Rab3* was not characterized further.

The other four tagged Rab3 variants were similarly assessed for constitutive activity. In each of these strains, slight (GFP-Rab3 and mCherry-Rab3) or no (3XFLAG-Rab3 and 2XHA-Rab3) signal was observed in the absence of excision (Figures S1B_2_, C_2_) or inversion (Figures S1D_2_, E_2_) above the signal seen in negative controls (Figures S1B_1_-E_1_) while robust signal was observed with pan-neuronal excision (Figures S1B_3_, C_3_) or inversion (Figure S1D_3_, E_3_). These latter four tagged Rab3 variants thus meet the necessary criteria of a conditional SV marker in that they are conditionally, and not constitutively, expressed.

### Assessment of synaptic vesicle localization

**Figure 2:**
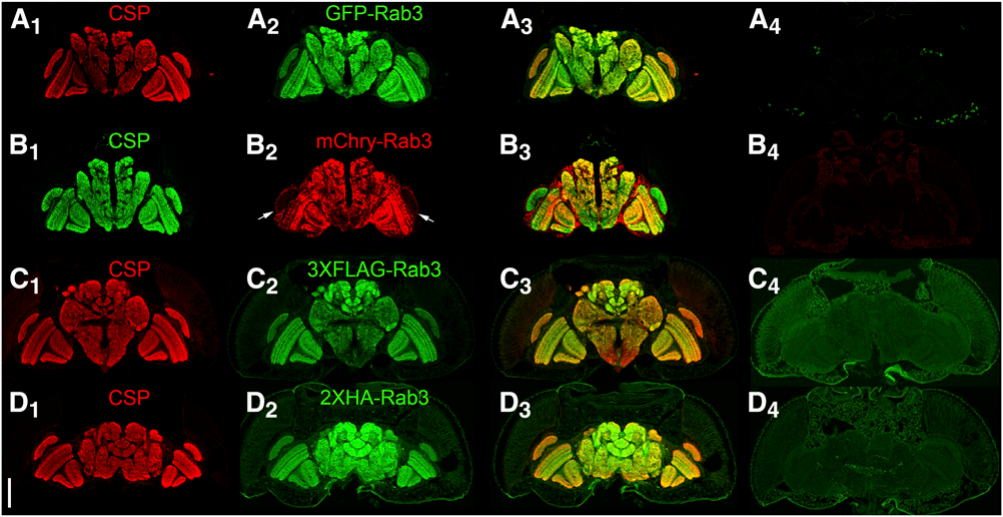
Assessment of co-localization between the endogenous synaptic vesicle marker Cysteine String Protein (CSP) and the tagged variants of Rab3 in immunostained cryostat sections of the adult brain after germline excision (A, B), or germline inversion (C, D). A_1_-D_1_) CSP localizes to the neuropil; A_2_) GFP-Rab3 shows a neuropil distribution; B_2_) mCherry-Rab3 shows a neuropil distribution except for very weak expression in the lamina (arrows); C_2_) 3XFLAG-Rab3 and D_2_) 2XHA-Rab3 show a neuropil distribution; A_3_-D_3_) Overlay. A_4_-D_4_) Controls without a GAL4 driver exhibit minimal levels of background fluorescence. A_4_-D_4_ were processed and imaged identically to A_2_-D_2_. Scale bar: 100μm.

In addition to conditional expression, an effective conditional SV marker must meet the criteria of strict localization to SVs. For an initial assessment, germline excision or inversion alleles of each of the four tagged variants of Rab3 were generated and assessed for co-localization with the endogenous SV-specific marker Cysteine String Protein (CSP) (Mastrogiacomo *et al.* 1994; Zinsmaier *et al.* 1994) in immuno-stained cryostat sections of adult heads. The distribution of the tagged Rab3 variants shows a general agreement with a neuropil localization (Figure 2A_2_-D_2_) and near precise overlap with CSP (Figures 2A_1_-D_1_) as is apparent in the overlay (Figures 2A_3_-D3). The one exception is that mCherry-Rab3 is very weak in the lamina (arrows, Figure 2B2). The low levels of background expression of the four tagged Rab3 variants in negative control adult brains should also be noted (Figures 2A_4_-D_4_).

**Figure 3:**
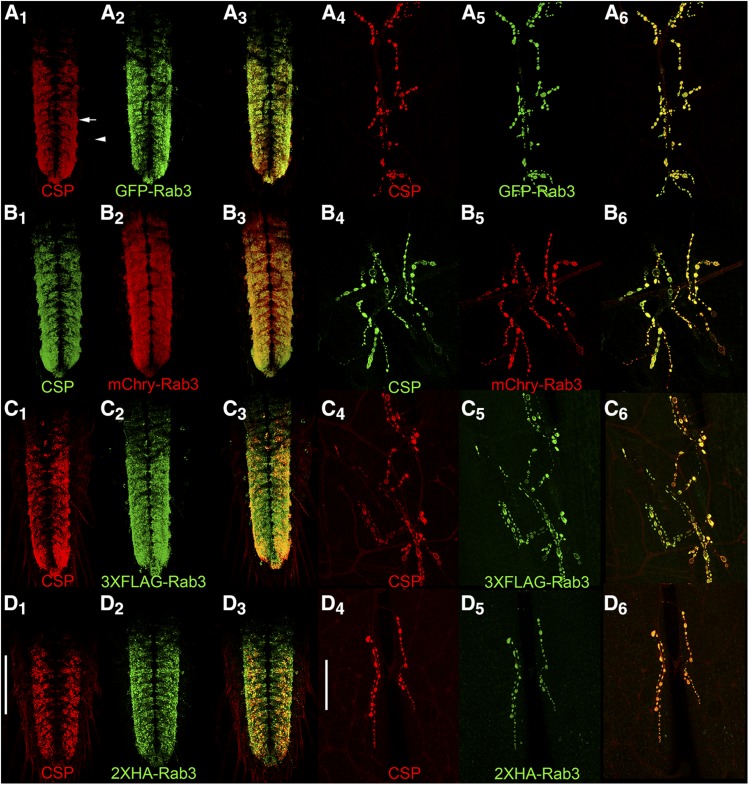
Assessment of co-localization via immunostaining between the endogenous synaptic vesicle marker Cysteine String Protein (CSP) and the tagged variants of Rab3 in third instar larva after pan-neuronal recombinase expression. In the ventral nerve cord (A_1-3_-D_1-3_) and at the neuromuscular junction (A_4-6_-D_4-6_), CSP exhibits a high degree of co-localization with GFP-Rab3 (A), mCherry-Rab3 (B), 3XFLAG-Rab3 (C), and 2XHA-Rab3 (D). Scale bars: 100μm (D_1_); 50μm (D_4_).

A comparison of the distribution of the four tagged Rab3 variants after pan-neuronal excision or inversion with endogenous CSP was also made in the larval VNC and at the larval neuromuscular junction (NMJ). In the larval VNC, all four tagged Rab3 variants exhibit nearly exclusive localization to the neuropil region (arrow, Figure 3A_1_) of the VNC (Figures 3, A_2_-D_2_) where synaptic terminals are located. Little or no localization was observed in the surrounding cortex (arrowhead, Figure 3A_1_) where the neuronal cell bodies reside (Prokop and Meinertzhagen 2006). A similar VNC distribution was observed for CSP (Figure 3A_1_-D_1_) that is highly co-incident with each of the tagged Rab3 variants as made apparent in the overlay (Figure 3A_3_-D_3_).

The co-localization of pan-neuronal expression of the tagged Rab3 variants with CSP was also assessed at the larval neuromuscular junction (NMJ). Each of the tagged Rab3 variants exhibits robust signal in pre-synaptic boutons at the larval NMJ (Figures 3A_5_-D_5_). CSP distributes similarly at the larval NMJ (Figures 3A_4_-D_4_) and this distribution is nearly identical to that of the tagged variants of Rab3 (Figures 3A_6_-D_6_). These results thus demonstrate that all four tagged variants of Rab3 exhibit the expected distribution of a synaptic vesicle-specific marker at the larval VNC and NMJ when pan-neuronally expressed. Taken together, these results in larva and adults showing that all four tagged Rab3 variants distribute co-incident with the established endogenous SV marker CSP are consistent with expectations for a SV marker.

### Phenotypic assessment of tagged Rab3 variants

Rab3 loss-of-function mutants exhibit the recessive phenotype at the third instar larval NMJ of altering the distribution of Brp (Drosophila homolog of mammalian ELKS/CAST/ERC) such that Brp forms a reduced number of puncta that are larger and more intensely labeled than in wildtype controls (Graf *et al.* 2009). To determine if the tagged Rab3 variants exhibit recessive or dominant phenotypes with respect to Brp expression, Brp immunostaining was performed in heterozygous and homozygous germline excision (*GFP-Rab3* and *mCherry-Rab3*) or germline inversion (*3XFLAG-Rab3* and *2XHA-Rab3*) alleles of the tagged Rab3 variants. Heterozygous and homozygous germline excisions of *GFP-Rab3* (Figure S2C_1_, D_1_) and *mCherry-Rab3* (Figure S2E_1_, F_1_) exhibit a similar Brp distribution as compared to controls (A) that is not indicative of the *rab3* loss-of-function phenotype (B). However, the levels of Brp expression are sharply reduced. Heterozygous and homozygous germline inversion alleles of *3XFLAG-Rab3* (Figure S2G_1_, H_1_) and *2XHA-Rab3* (Figure S2I_1_, J_1_) also exhibit a similar Brp distribution as compared to controls that is not reminiscent of the rab3 loss-of-function phenotype. In both germline inversion alleles there is a perceptible, but less significant, reduction in Brp expression levels than in the *GFP-Rab3* or *mCherry-Rab3* germline excision alleles. All NMJs were co-labeled for their corresponding tagged Rab3 variants as shown to the right of each Brp image (C_2_-J_2_). Since the reduced levels of Brp phenotype is observed in both the heterozygous and homozygous conditions, the phenotype is dominant. The simplest explanation for this phenotype is that the fluorescent and epitope tags fused to Rab3 sterically hinder the formation of the five-protein oligomeric complex comprising the core of pre-synaptic active zones that includes Brp and Rab3-Interacting Molecule/RIM (which directly interacts with Rab3) (Südhof 2012), thus destabilizing all of the proteins in the complex including Brp. Despite the dominant Brp expression phenotype, all four of the germline excision or germline inversion alleles of the tagged Rab3 variants are homozygous viable and fertile with no obvious locomotor or behavioral defects.

### Comparison to existing Drosophila SV markers

To assess the comparative reliability of conditional GFP-Rab3 and mCherry-Rab3 as SV markers with existing UAS-Neuronal-synaptobrevin-GFP (*UAS-N-syb-GFP*) and UAS-Synaptotagmin-GFP (*UAS-Syt-GFP*) SV markers that have long been used to detect SVs in Drosophila (Zhang *et al.* 2002), each was pan-neuronally expressed and their distributions assessed in the third instar larval VNC. The same pan-neuronal *N-syb-GAL4* driver was used for excision of the STOP cassettes from *B2RT-STOP-B2RT-GFP-Rab3* and *B2RT-STOP-B2RT-mCherry-Rab3* that were used for expression of N-syb-GFP and Syt-GFP. Pan-neuronal expression of GFP-Rab3 (Figure S3A) and mCherry-Rab3 (Figure S3B) reveal that their distributions are restricted to the neuropil region of the VNC where SVs are known to localize (Prokop and Meinertzhagen 2006). In contrast, a significant fraction of both N-syb-GFP (Figure S3C) and Syt-GFP (Figure S3D) localizes to neuronal cell bodies as revealed in both the dorsal (top) and orthogonal (bottom) views (arrowheads, Figures S3C, D) as well as to the lateral nerves emanating from the VNC. No neuronal cell body or lateral nerve signal was observed in immunostains for the endogenous SV-specific proteins Syt (Figure S3E) or Synapsin (Syn) (Figure S3F), thus indicating the cell body and lateral nerve signals observed with pan-neuronal expression of N-syb-GFP and Syt-GFP is spurious and is not representative of actual SVs. The highly similar distributions of GFP-Rab3 and mCherry-Rab3 as compared to the endogenous SV proteins Syt and Syn demonstrate GFP-Rab3 and mCherry-Rab3 are significantly more reliable markers of SVs than UAS-N-syb-GFP or UAS-Syt-GFP.

### Assessment of SV localization and sensitivity in specific neuron subtypes

With conditionality and pan-neuronal synaptic vesicle localization thus established, the remaining criteria an effective conditional SV marker must satisfy are that its sensitivity must be high enough to be detected in individual neurons and that it localizes to SVs in individual neurons. To make these assessments, three diverse cholinergic neuron types and one dopaminergic neuron type were chosen for analysis: larval Basin-4 neurons (Ohyama *et al.* 2015), adult L2 lamina neurons (Tuthill *et al.* 2013), adult mushroom body Kenyon cell γ neurons (Aso *et al.* 2014), and adult mushroom body PPL1-α3 neurons (Aso *et al.* 2014). Cholinergic neuron types were predominantly chosen so the conditional cholinergic SV marker HA-vAChT (Pankova and Borst 2017) could be used to assess SV localization of the tagged Rab3 variants.

### Larval Basin-4 neurons

**Figure 4:**
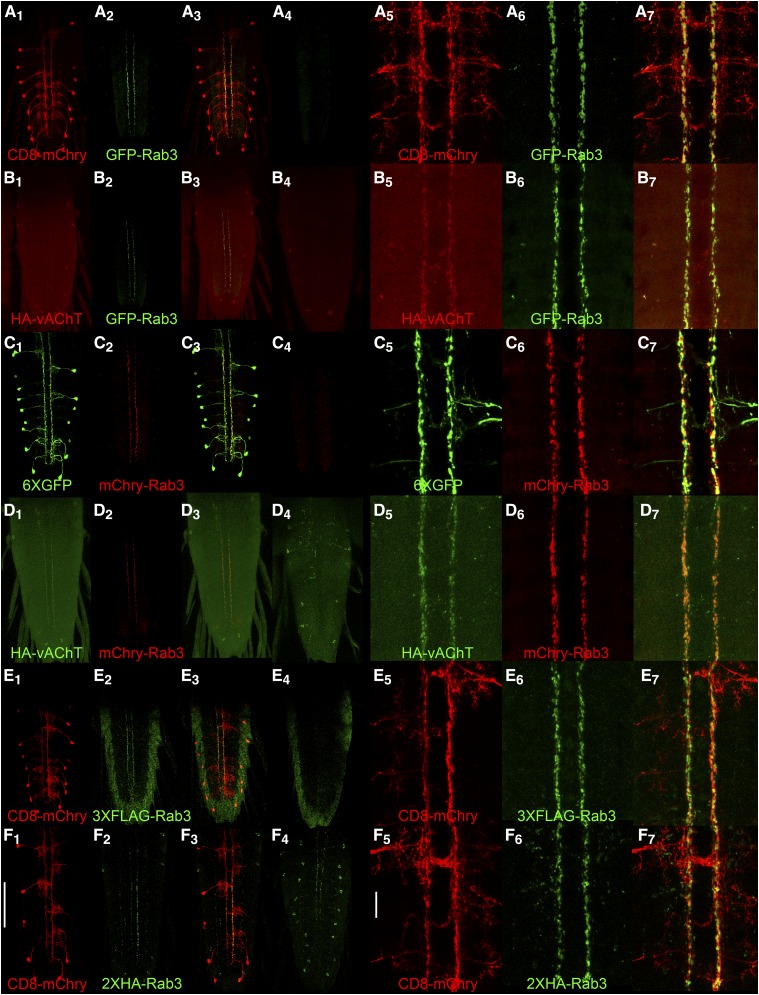
Conditional expression of tagged Rab3 variants in Basin-4 larval neurons. A_1_, C_1_, E_1_, F_1_) The location and structure of Basin-4 neurons in the larval ventral nerve cord visualized with the plasma membrane marker CD8-mcherry or the cytoplasmic marker 6XGFP. There are two symmetric Basin-4 neurons per segment with peripherally located cell bodies and branched axodendritic processes extending medially to the central region of the VNC. A_2_, C_2_, E_2_, F_2_) Each of the tagged Rab3 variants exhibits highly restricted localization to two longitudinal strips near the center of the VNC when conditionally expressed in Basin-4 neurons. A_3_, C_3_, E_3_, F_3_) Overlay. A_4_, C_4_) Background levels of expression of GFP-Rab3 and mCherry-Rab3 in the absence of a GAL4 driver. A_4_ and C_4_ were imaged and processed identically to A_2_ and C_2_, respectively. E_4_, F_4_) Background levels of signal detected with anti-FLAG and anti-HA antibodies in negative controls. E_4_ and F_4_ were imaged and processed identically to E_2_ and F_2_, respectively. A_5_, C_5_, E_5_, F_5_) High magnification images of the central region of the VNC of Basin-4 neurons visualized with CD8-mCherry or 6XGFP. A_6_, C_6_, E_6_, F_6_) High magnification images of the tagged Rab3 variants in the central region of the VNC of Basin-4 neurons. A_7_, C_7_, E_7_, F_7_) Overlay. The tagged Rab3 variants are excluded from all regions of Basin-4 neurons except for two central longitudinal strips. B_1_, D_1_) The cholinergic synaptic vesicle marker HA-vAChT localizes to two central strips in the VNC upon conditional expression in Basin-4 neurons. B_2_, D_2_) GFP-Rab3 and mCherry-Rab3 localization is restricted to two central strips in the VNC upon conditional expression in Basin-4 neurons. B_3_, D_3_) Overlay. HA-vAChT co-localizes with GFP-Rab3 and mCherry-Rab3 in two central strips of Basin-4 neurons. B_4_, D_4_) Background levels of expression of HA-vAChT in the absence of a GAL4 driver. B_4_ and D_4_ were imaged and processed identically to B_2_ and D_2_, respectively. B_5_, D_5_) High magnification images of of HA-vAChT conditional expression in Basin-4 neurons in the central region of the VNC. B_6_, D_6_) High magnification images of of GFP-Rab3 and mCherry-Rab3 conditional expression in Basin-4 neurons in the central region of the VNC. B_7_, D_7_) Overlay. HA-vAChT exhibits nearly indistinguishable localization with GFP-Rab3 and mCherry-Rab3 in Basin-4 neurons at high magnification. Scale bars: 100μm (F_1_); 15μm (F_5_).

Basin-4 neurons are located in the larval VNC and consist of one symmetric pair per segment with cell bodies near the periphery and highly branched axodendritic processes that project to the midline (low magnification-Figures 4A_1_, C_1_, E_1_, F_1_; high magnification-Figures 4A_5_, C_5_, E_5_, F_5_). In contrast to the CD8-mCherry plasma membrane and 6XGFP cytoplasmic markers that distribute throughout Basin-4 neurons, the distribution of all four tagged variants of Rab3 is spatially restricted to two thin longitudinal strips of expression near the midline (low magnification-Figures 4A_2_, C_2_, E_2_, F_2_; high magnification-Figures 4A_6_, C_6_, E_6_, F_6_). These strips of expression were not observed in controls containing all genetic components except the Basin-4 GAL4 driver (Figures 4A_4_, C_4_, E_4_, F_4_). The difference in distribution between CD8-mCherry and 6XGFP that highlight the entire neuron and the tagged Rab3 SV markers is also clearly apparent in the overlays (low magnification-Figures 4A_3_, C_3_, E_3_, F_3_; high magnification-A_7_, C_7_, E_7_, F_7_).

To determine if the distribution of the tagged Rab3 variants in thin longitudinal stripes near the midline represents *bona fide* synaptic vesicles, HA-vAChT was co-conditionally expressed with GFP-Rab3 or mCherry Rab3 in Basin-4 neurons. Like GFP-Rab3 (low magnification-Figure 4B_2_; high magnification Figure 4B_6_) and mCherry-Rab3 (low magnification-Figure 4D_2_; high magnification-Figure 4D_6_), HA-vAChT also localizes to two thin strips near the midline of the VNC (low magnification-Figures 4B_1_, D_1_; high magnification-Figures 4B_5_, D_5_). The strips of HA-vAChT expression show a near exact coincidence, especially evident at high magnification, with the strips of GFP-Rab3 (low magnification-Figure 4B_3_; high magnification-Figure 4B_7_) and mCherry-Rab3 (low magnification-Figure 4D_3_; high magnification-4D_7_). These results demonstrate GFP-Rab3 and mCherry-Rab3 are reliable markers of SVs in Basin-4 neurons. It was not possible to do the comparison of conditional HA-vAChT with either 3XFLAG or 2XHA in Basin-4 or the other neuron types described below because each of these markers relies on potentially cross-reactive FRTs for conditional expression. Nevertheless, given the similarity of the 3XFLAG and 2XHA distribution patterns to HA-vAChT, it is only reasonable to infer their validity as SV markers in Basin-4 neurons as well.

### Visual system L2 lamina neurons

**Figure 5:**
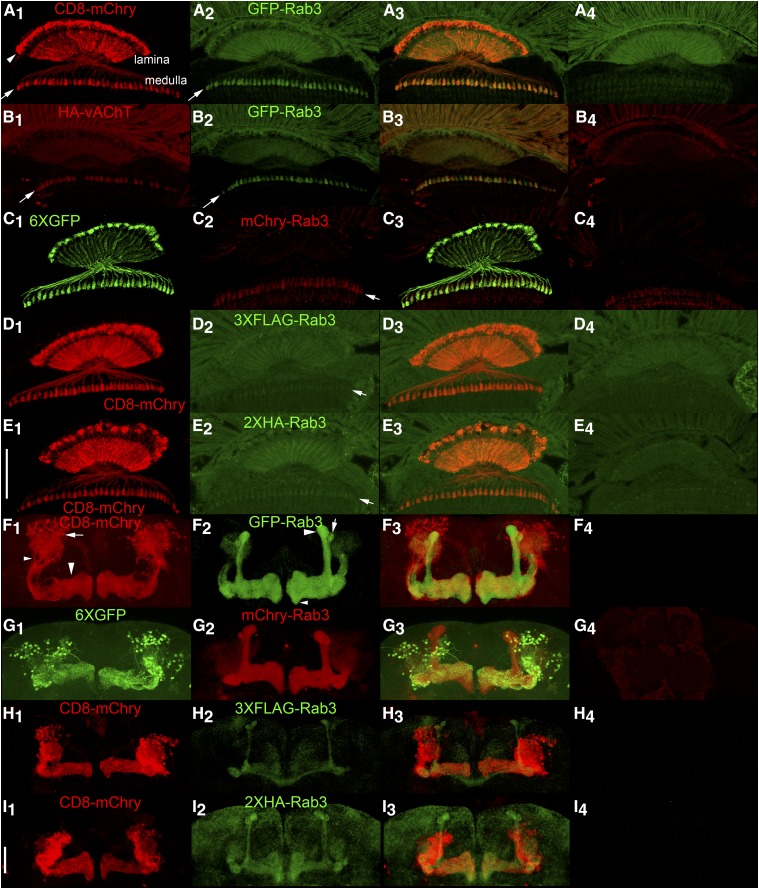
Conditional expression of tagged Rab3 variants in adult L2 lamina optic lobe neurons and MB131B adult γ-lobe mushroom body neurons. A_1_, C_1_, D_1_, E_1_) Anatomy of L2 lamina neurons visualized with the plasma membrane marker CD8-mCherry or the cytoplasmic marker 6XGFP. The structure of L2 lamina neurons includes cell bodies located between photoreceptor rhabdomeres and the lamina neuropil (arrowhead, A_1_), axodendritic processes that extend through the lamina neuropil, and synaptic terminals in the medulla (arrow, A_1_). A_2_, C_2_, D_2_, E_2_) Localization of tagged Rab3 variants in L2 lamina neurons. Arrows indicate synaptic terminals in the medulla. A_3_, C_3_, D_3_, E_3_) Overlay. A_4_, C_4_, D_4_, E_4_) Controls for the tagged Rab3 variants in the absence of a GAL4 driver showing background levels of autofluorescence due to w+ pigment present in photoreceptors. The significant levels of autofluorescence in the lamina, especially in the green channel, confound assessment of localization in the lamina neuropil. Signal above background is detectable in L2 synaptic terminals for all four tagged Rab3 variants, although it is noticeably weaker for 3XFLAG-Rab3 and HA-Rab3. B_1-3_) Conditional co-expression of HA-vAChT (B_1_) and GFP-Rab3 (B_2_) in L2 lamina neurons. Arrows indicate synaptic terminals. B_3_) Overlay. HA-vAChT and GFP-Rab3 show a high degree of co-localization in L2 synaptic terminals. B_4_) Control for HA-vAChT in the absence of a GAL4 driver. F_1_, G_1_, H_1_, I_1_). Anatomy of MB131B Kenyon cell neurons γ-lobe mushroom body neurons visualized with CD8-mCherry or 6XGFP. For one lobe in F_1_, cell bodies are indicated with an arrow, axodendritic processes with a small arrowhead, and γ-lobe neuropil with a large arrowhead. F_2_, G_2_, H_2_, I_2_) Localization of tagged Rab3 variants in MB131B mushroom body neurons. F_3_, G_3_, H_3_, I_3_) Overlay. All tagged Rab3 variants localize to the γ-lobe neuropil where synaptic terminals are located, but not to the cell body or axodendritic regions of MB131B neurons with GFP-Rab3 and mCherry-Rab3 showing significantly stronger signal than 3XFLAG-Rab3 or 2XHA-Rab3. In addition, all tagged variants localize to mushroom body α- and α’-lobe neuropil and variably in β-lobe neuropil. In F_2_ for one lobe the α-lobe is indicated with a large arrowhead, the α’-lobe with an arrow and the β-lobe with a small arrowhead. F_4_, G_4_, H_4_, I_4_) Controls for the tagged Rab3 variants in the absence of a GAL4 driver imaged and processed identically to their corresponding variants shown in F_2_-I_2_. The absence of signal in mushroom body neuropil in F_4_-I_4_ suggests the signal in mushroom body neuropil outside the γ-lobe in F_2_-I_2_ is due to developmental expression of the MB131B GAL4 driver in neurons that comprise the other neuropils and is not due to constitutive/leaky expression from the tagged Rab3 variants. Scale bars: 50μm (E_1_ and I_1_).

L2 lamina neurons (Figures 5A_1_, C_1_, D_1_, E_1_) are present in each of the ∼800 ommatidia of the fly eye and have cell bodies (arrowhead, Figure 5A_1_) located between the photoreceptor rhabdomeres and the lamina neuropil (Prokop and Meinertzhagen 2006). L2 lamina neurons extend axodendritic processes through the lamina neuropil and into the medulla neuropil where their synaptic terminals are located (arrow, Figure 5A1). Conditional expression of the tagged Rab3 variants in L2 lamina neurons reveals a horizontal stripe of expression (arrows-Figures 5A_2_, C_2_, D_2_, E_2_) coincident with L2 pre-synaptic terminals and above background levels of fluorescence observed in the medulla of controls (Figures 5A_4_, C_4_, D_4_, E_4_). These results indicate all four tagged Rab3 variants localize to the pre-synaptic terminals of L2 neurons. However, due to autofluorescence in the lamina from the w+ eye pigment used as a marker in transgenes (Figures 5A4, C4, D4, E4) it is not possible to conclude one way or the other if the tagged Rab3 variants localize to L2 cell bodies or L2 axodendritic processes in the lamina as autofluorescence and legitimate signal cannot be distinguished in these regions. The apparent overlap in fluorescence seen in these regions (Figures 5A_3_, C_3_, D_3_, E_3_) is thus not interpretable. It is also apparent from these experiments that the signal from GFP-Rab3 and mCherry-Rab3 is significantly stronger than that of either 3XFLAG-Rab3 or 2XHA-Rab3.

A comparison of the distribution of the cholinergic SV marker HA-vAChT (Figure 5B_1_) with GFP-Rab3 (Figure 5B_2_) upon co-conditional expression in L2 lamina neurons revealed a highly overlapping distribution in L2 pre-synaptic terminals in the medulla (Figure 5B_3_). This accumulation of HA-vAChT in L2 pre-synaptic terminals in the medulla confirms these terminals as the region of L2 neurons where SVs localize and the accumulation of all four tagged Rab3 variants at these terminals is consistent with their authenticity as a SV marker. It was not possible to compare mCherry-Rab3 with HA-vAChT in a similar experiment in L2 lamina neurons because the *HA-vAChT* chromosome carries a 3X3P-DsRed marker that is expressed in all photoreceptors, including photoreceptors R7 and R8 that project their axons to the same region of the medulla as the L2 lamina neuron, and antibodies to mCherry also recognize the DsRed protein from which it was derived (*i.e.*, mCherry-Rab3 and the DsRed marker from *HA-vAChT* cannot be unambiguously distinguished in the medulla).

### Mushroom body γ neurons

Mushroom body γ neurons (Figures 5F_1_, G_1_, H_1_, I_1_), represented by the MB131B GAL4 driver, are a subset of ∼600 mushroom body Kenyon cells with dorsally located cell bodies (arrow, F_1_) that project axodendritic process downward (small arrowhead, F_1_) to the γ-lobe of the mushroom body (large arrowhead, F_1_) where they make synaptic connections (Tanaka *et al.* 2008; Pech *et al.* 2013; Aso *et al.* 2014). Consistent with expectations, the tagged Rab3 variants distribute to the γ-lobe neuropil of the MB upon conditional expression in MB131B neurons (Figures 5F_2_, G_2_, H_2_, and I_2_) but not to the cell body or axodendritic processes (Figures 5F_3_, G_3_, H_3_, I_3_). Unexpectedly, in addition to the distribution to the γ-lobe, the tagged Rab3 variants also distribute to the α- (large arrowhead, Figure 5F2), α’- (arrow, Figure 5F_2_), and β- (small arrowhead, Figure 5F_2_) lobes of the MB. Since no expression in the mushroom body was observed in controls of any of the four tagged Rab3 variants (Figures 5F_4_, G_4_, H_4_, I_4_) this expression cannot be attributed to constitutive/leaky expression of the tagged Rab3 variants. The most plausible explanation for the observed expression in other lobes of the mushroom body besides the γ-lobe is that the MB131B GAL4 driver is historically active earlier in development in the MB neurons that in adulthood comprise the α-, α’-, and β-lobes. In this scenario, irreversible recombination events occur in those neurons during development even though by adulthood the expression of the MB131B GAL4 driver has become restricted to the subset of Kenyon cells that comprise the γ-lobe. Regardless of the explanation, the important finding in this assessment is that the tagged variants of Rab3 are detectable and localize exclusively to the neuropil region of the lobes of the MB where pre-synaptic terminals and synaptic vesicles are located and not to cell bodies or axodendritic regions where SVs would not be expected. As in the L2 lamina neurons, signal from 3XFLAG-Rab3 and 2XHA-Rab3 is clearly weaker than for GFP-Rab3 and mCherry-Rab3 in the MB neuropil.

Co-localization of HA-vAChT with GFP-Rab3 and mCherry-Rab3 using the MB131B GAL4 driver was also attempted in co-conditional expression experiments. Although HA-vAChT signal was weak (Figures S4A_1_, B_1_) MB neuropil signal above background levels in controls (Figures S4A_4_, B_4_) was discernable in HA-vAChT experimental genotypes and appeared to strongly coincide with the much stronger expression of GFP-Rab3 (Figures S4A_2_, A_3_) and mCherry-Rab3 (Figures S4B_2_, B_3_). This co-localization result also supports the validity of GFP-Rab3 and mCherry-Rab3 as SV markers, despite sub-optimal HA-vAChT expression. The localization of the independently developed conditional HA-vAChT in other lobes of the MB besides the γ-lobe also supports the explanation of historical developmental expression of the MB131B driver in other MB neurons.

### Mushroom body PPL1-α3 neurons

**Figure 6:**
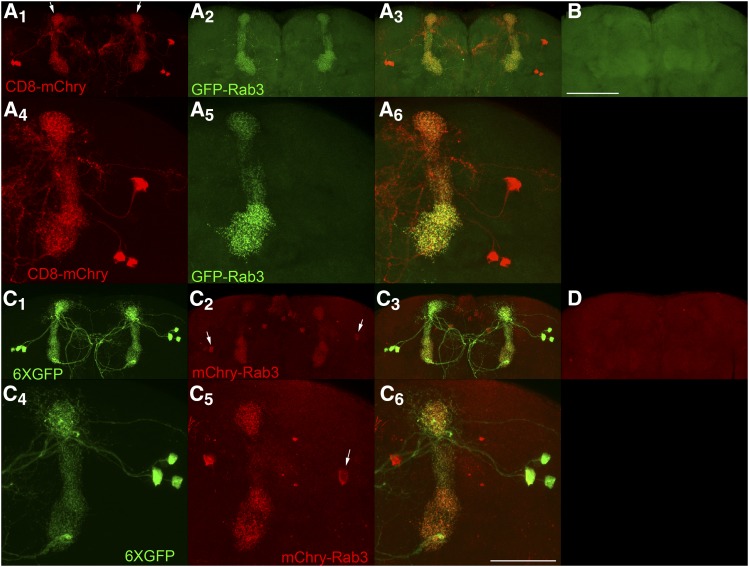
Conditional expression of GFP-Rab3 and mCherry-Rab3 in dopaminergic PPL1-α3 neurons. A_1_, A_4_, C_1_, C_4_) The PPL1-α3 neurons are represented by the MB060B GAL4 driver that expresses in three neurons of each brain hemisphere as visualized with the plasma membrane marker CD8-mCherry or the cytoplasmic marker 6XGFP. PPL1-α3 neurons extend axodendritic processes that project medially from their cell bodies primarily to the α-lobe of the mushroom body (arrows, A_1_), but also to the midline. A_2_, A_5_) Conditional expression of GFP-Rab3 in PPL1-α3 neurons. GFP-Rab3 localizes exclusively to the α-lobe of the mushroom body where synaptic contact sites are located with no discernible expression in cell bodies or processes outside the α-lobe. A_5_) A higher resolution image reveals the fine scale structure of SV distribution. A_3_, A_6_) Overlays. B) Negative control with all genetic components except the GAL4 driver shows background levels of signal. C_2_, C_5_) Conditional expression of mCherry-Rab3 in PPL1-α3 neurons. mCherry-Rab3 localizes predominantly to the α-lobe of the mushroom body, although cell body signal in some PPL1-α3 neurons is perceptible (arrows). This likely does not represent actual SVs since no cell body signal was observed with GFP-Rab3. Outside the cell bodies, mCherry-Rab3 localizes exclusively to the α-lobe region of the mushroom body in a distribution almost indistinguishable from GFP-Rab3. C_5_) A higher resolution image shows the fine scale structure of SV localization. C_3_, C_6_) Overlays. D) Negative control with all genetic components except the GAL4 driver shows background levels of signal. These results demonstrate the signal from the GFP-Rab3 and mCherry-Rab3 SV markers is of sufficient strength to reveal the SV distribution in individual neurons. Scale bars 100μm (B); 50μm (C_6_).

Lastly, the sensitivity of detection of GFP-Rab3 and mCherry-Rab3 was assessed in dopaminergic PPL1-α3 neurons. This neuron type is represented by the MB060B GAL4 driver (Aso *et al.* 2014) and expresses in three neurons per brain hemisphere. PPL1-α3 neurons have lateral cell bodies that project medially to the vertical α lobes (arrows, Figure 6A_1_) with axodendritic processes extending to the midline (Figures 6A_1_, C_1_). Conditional expression of GFP-Rab3 in these neurons reveals exclusive localization to the vertical mushroom body α-lobe (Figure 6A_2_) where the mushroom body neuropil and SVs are located. No expression of GFP-Rab3 was detected in controls in which the GAL4 driver was not present (Figure 6B). Higher resolution images reveal the fine scale structure of SV distribution in PPL1-α3 neurons (Figure 6A_5_). Conditional expression of mCherry-Rab3 in these neurons reveals a predominant distribution to the mushroom body α lobes, although mCherry-Rab3 is discernable in the cell bodies of some PPL1-α3 neurons (arrows Figures 6C_2_, C_5_) that likely does not reflect actual SVs since no GFP-Rab3 was observed in the cell bodies of this neuron type. The small amount of mCherry-Rab3 detectable in some PPL1-α3 neuronal cell bodies may reflect less efficient trafficking of mCherry-Rab3 through the ER/Golgi as compared to GFP-Rab3. However, like GFP-Rab3, no mCherry-Rab3 was observed in the axodendritic processes outside the α lobe, thus indicating that once mCherry-Rab3 exits the cell body it traffics with similar efficiently as GFP-Rab3 to sites of SV release. mCherry-Rab3 was not detectable in controls lacking a GAL4 driver (Figure 6D). The fine scale structure of SV localization can be discerned in higher resolution images (Figure 6C_5_). As was demonstrated with larval Basin-4 neurons, these results with adult PPL1-α3 neurons establish that the GFP-Rab3 and mCherry-Rab3 SV markers are sensitive enough to reveal the distribution of SVs in individual neurons.

## Discussion

The design and characterization of four conditional tagged Rab3 SV markers for Drosophila has been described. These SV markers have been characterized for conditionality, specificity for SV localization (pan-neuronally and in four diverse neuron sub-types), and sensitivity. Data has been presented for all four SV markers that convincingly demonstrates conditional expression and highly specific localization to SVs. A significant difference in sensitivity of detection was, however, evident between the various SV markers. The GFP-Rab3 and mCherry-Rab3 SV markers exhibited noticeably stronger signal, especially in adults, than did 3XFLAG-Rab3 or 2XHA-Rab3, or the previously reported HA-vAChT. The reasons for this are uncertain, but factors that could play a role include differences in the affinity of primary antibodies, differences in protein stability, and a differential effect on transcription of the upstream *B2RT* or *FRT* remaining after excision or inversion. Due to their higher sensitivity, the GFP-Rab3 or mCherry-Rab3 SV markers are thus probably the better choice for most experiments unless additional potentially cross-reacting *B2RTs* are present that could confound the experiment.

The SV markers reported here should thus be useful for a variety of purposes for understanding neuronal communication and ultimately how neural circuits generate behavior. The only limitation is the availability of specific driver lines to drive B2 or FLP recombinase expression in small neuronal subsets or single neuron types. Hundreds of GAL4 or split-GAL4 drivers currently exist with specificity for specific neuron types (Jenett *et al.* 2012; Manning *et al.* 2012; Tuthill *et al.* 2013; Aso *et al.* 2014; Li *et al.* 2014; Jovanic *et al.* 2016; Wu *et al.* 2016; Wolff and Rubin 2018) whose SV distribution could be determined with the tagged Rab3 variants described herein. There is also the potential to develop additional novel GAL4 drivers for most any neuron type of interest (Dionne *et al.* 2018) and the number of such drivers will only increase over time. In addition to defining the region of a neuron where SVs reside (and thereby where within a neuron SV fusion/neurotransmitter release can occur) other potential uses for these SV markers include immuno-isolation of SVs from specific neuron sub-types, and, for the endogenously fluorescent GFP-Rab3 and mCherry-Rab3, studies of the dynamics of SV movement in living neurons.
